# IS THIS (TELE)WORKING? A path model analysis of the relationship between telework, job demands and job resources, and sustainable employability

**DOI:** 10.3233/WOR-240033

**Published:** 2025-03-18

**Authors:** Elizabeth M. Beekman, Madelon M.L. Van Hooff, Krisna Adiasto, Brigitte J.C. Claessens, Beatrice I.J.M. Van der Heijden

**Affiliations:** a Behavioural Science Institute, Radboud University, Nijmegen, The Netherlands; b Faculty of Psychology, Open Universiteit, Heerlen, The Netherlands; c Institute for Management Research, Radboud University, School of Management, Nijmegen, The Netherlands; d Open Universiteit, Heerlen, The Netherlands; e Research Group HRM and Organizational Behavior, Ghent University, Ghent, Belgium; f Hubei Business School, Hubei University, Wuhan, China; g Kingston Business School, Kingston University, London, UK

**Keywords:** Remote work, work from home, sustainable employability, Job Demands-Resources model, work pressure, social support, workplace communication, role clarity

## Abstract

**BACKGROUND:**

Technological advancements and the COVID-19 crisis have accelerated the adoption of telework, impacting employees’ work dynamics. Moreover, an aging workforce emphasises the need for sustainable employability. With reference to the Job Demands-Resources (JD-R) Model, this study explores how telework relates to job demands and job resources and, subsequently, to sustainable employability.

**OBJECTIVE:**

The present study investigates the repercussions of increased telework on employees’ sustainable employability. Hypotheses posit direct and indirect (i.e., mediated) relationships, providing insights for evidence-based telework policies.

**METHODS:**

Data from 552 government employees was collected through an online survey. Data collection occurred during the COVID-19 pandemic when widespread teleworking was prevalent. A path model was employed to analyse associations between telework, job demands (specifically work pressure), job resources (social support, workplace communication, and role clarity), and sustainable employability dimensions, including vitality, work ability, and employability.

**RESULTS:**

Our path model reveals that heightened telework was associated with elevated work pressure and diminished role clarity. Surprisingly, a positive association emerges between work pressure and sustainable employability. Notably, no significant relationship is found between telework, social support, and workplace communication. Role clarity is likely to be pivotal, positively influencing vitality and employability.

**CONCLUSIONS:**

This study provides valuable insights into the effects of telework on job demands, resources, and sustainable employability. The unexpected positive association between work pressure and sustainable employability challenges conventional stressor paradigms. The relationships between telework, job demands, job resources, and sustainable employability uncovered in this study can contribute to evidence-based teleworking policies and strategies that support employee health and employability amidst evolving work structures.

## 1 Introduction

Technological advancements have paved the way for New Ways of Working [1]: changes in both the content and organisation of work, including time- and place-independent work. These developments have caused many employees to begin (partially) teleworking, i.e., working from a remote location that is not the organisation’s centralised workspace [2]. The COVID-19 crisis has further expedited the development of possibilities for, and organisational structures in support of, telework [3]. The rapid implementation of telework has given rise to questions regarding its effects on employees, as well as the possible benefits and risks of telework for organisations [4, 5]. Large groups of organisations have held on to telework, even post-COVID restrictions, whereas others have opted to shift back to (mostly) working in the office [6, 7].

Simultaneously, in the Netherlands – where the current study took place – consequences of the ageing working population (e.g., foreseeable personnel shortages) have led to changes in national policy, heightening the legal retirement age from 65 to the age of 67 [8]. Employees are thus expected to work up to an older age, highlighting the need to keep employees happy, healthy, and productive [9], and thus prioritize their sustainable employability [10]. The context of telework can influence important work factors that may be significant for employees’ sustainable employability (i.e., employees’ capacities to function in work and on the labour market throughout their working lives [11]). For instance, a study by Collins and associates [12] examined how social support can lack in situations of teleworking. They furthermore noted how this lack of social support can negatively affect employees’ development and opportunities for growth within the organisation, thus affecting their employability [10]. In a similar vein, Wöhrmann and Ebner [13], examining the bright and dark sides of telework, found indirect relationships between telework and employee health via working time control, time pressure, boundaryless working hours, relationships with co-workers, and disturbances and interruptions, herewith supporting the notion of an indirect link between telework and sustainable employability.

This impact of changing work factors in the context of telework and sustainable employability can be understood through the lens of the Job Demands-Resources (JD-R) Model [14, 15]. The JD-R model [14] proposes that every job possesses its own specific risk determinants associated with job stress, and that these determinants can be classified in two general categories, namely job demands and job resources. Job demands are described as physical, psychological, social, or organisational aspects of a job that require cognitive or emotional effort. Meanwhile, job resources are described as physical, psychological, social, or organisational aspects of a job that reduce the physical and/or emotional effort required to handle job demands. Following the key notions underlying the JD-R model, it is relevant to note that job demands are not necessarily negative. Rather, job demands create strain only when their fulfilment requires high effort, which employees cannot adequately recover from [16]. On the other hand, job resources are valued as being important means to either cope with job demands, or simply means to the achievement or protection of other valued resources. The presence of job resources is thus expected to enhance employees’ motivation [14]. In addition, the presence of job resources can contribute to reducing the risk of burnout, and consequently, sick leave [17, 18].

Thus, the JD-R model is built upon two underlying psychological processes that play a role in the development of job strain and motivation [14, 15]. The first comprises a so-called health impairment process. This is a situation in which high job demands (such as the amount of perceived work pressure, which we posit to be related to telework) exhaust employees’ mental and physical resources, and may therefore lead to exhaustion and health problems [14, 19]. The second underlying process is motivational in nature, and posits that job resources (such as the amount of perceived social support, which we argue to be related to telework [12]) have either intrinsic (i.e., they foster growth, learning and development) or extrinsic (i.e., they are instrumental in achieving work goals) motivational potential. Both these motivational potentials lead to positive work outcomes, such as high work engagement and job performance [14, 15]. Job resources are thus essential to deal with job demands, but they are also rewarding in and of themselves, as they help fulfill basic human needs such as those for autonomy, relatedness, and competence [15, 20]. Ample research provides evidence for the JD-R model’s dual pathways to employee well-being, and further demonstrates that job demands and job resources can predict important organisational outcomes (see Bakker and colleagues [15] for an extensive overview).

Using the framework of the JD-R model, previous studies have examined the effects of teleworking on outcomes such as psychological well-being and emotional exhaustion [21–23]. For instance, a study conducted by Bilotta and colleagues stresses that telework in the COVID-19 context resulted in additional cognitive and emotional demands, such as role ambiguity and spillover of work-life boundaries [21] Yet, despite this seminal work, the full extent of the relationship between teleworking and sustainable employability, particularly through factors in the JD-R model, remains understudied. Here, it is relevant to note that employees do experience a change in their job demands and job resources during telework [19], which we posit could relate to their sustainable employability.

### 1.1 The present study

Based on the above, the aim of the current study is to explore the possible relationship between teleworking and sustainable employability through the changing of available job demands and job resources. We focused on the job demand ‘work pressure’ and the job resources ‘social support’, ‘workplace communication’, and ‘role clarity’; these demands and resources and their relationship to sustainable employability are detailed in later paragraphs. Finding out more about the job demands and job resources that are in play in the setting of teleworking, along with how these factors relate to employees’ sustainable employability, may offer significant theoretical grounds to facilitate the creation of evidence-based teleworking policies. Furthermore, the current study may help shed light on the ways in which employees who telework can be supported, with the aim of protecting and enhancing their employability. To this end, the following research question was formulated: “Does teleworking influence perceived job demands and job resources among employees, and how do these job demands and job resources relate to perceived sustainable employability?”

The current study was conducted during the COVID-19 pandemic when a substantial number of employees were teleworking. The employer-employee relationship changed in a radical manner due to this pandemic, as most organisations were obliged to enact work from home (i.e., telework) policies in an attempt to prevent the spread of COVID-19 [24]. The concept of telework is not new and has already received attention in scholarly work [25, 26]. Despite this, telework during the COVID-19 pandemic is unique in that employees were forced to work from home, rather than telework being a voluntary alternative to work from the office. Crucially, the pandemic acted as a catalyst for many organisations to make telework the new normal [3], further stressing the need to increase our understanding of its possible relation to employees’ sustainable employability.

### 1.2 Sustainable employability

Sustainable employability generally refers to employees’ capacities to function in work throughout their working life [27]. A more comprehensive definition of sustainable employability is provided by Van der Klink and associates [11], who state that: “Sustainable employability means that throughout their working lives, workers can achieve tangible opportunities in the form of a set of capabilities. They also enjoy the necessary conditions that allow them to make a valuable contribution through their work, now and in the future, while safeguarding their health and welfare. This requires, on the one hand, a work context that facilitates this for them and, on the other, the attitude and motivation to exploit these opportunities” (p. 74). This definition underscores that sustainable employability is the result of the interaction between the individual and their work.

In their exemplary review on sustainable employability, Jabeen and associates [28] broadly describe sustainable employability as a unique combination of an individual’s vitality (i.e., having the motivation and resilience to complete one’s work responsibilities), work ability (i.e., being physically and mentally able to perform one’s work), and employability (i.e., having the necessary skills, knowledge and competence to perform one’s work) [11, 29, 30]. Schaufeli and Bakker [31] define vitality as the ability to work in an energetic, resilient, fit, and tireless manner with great perseverance. Vitality is thus central to an individual’s capacity to effectively perform essential roles and responsibilities. Next, Ilmarinen and colleagues [32] define work ability as the extent to which employees are physically, mentally, and socially capable of working. Lastly, Van der Heijde and Van der Heijden [33] define employability as an individual’s ability to function well in their position, to make progress in their career, and to find a (different) job if that is necessary. Highly employable individuals possess the capacity to keep, obtain, and create work by optimally utilising and developing their competencies. As such, their chances of retaining their present positions are high [34]. Taken together, employees who are sustainably employable thus demonstrate high vitality, high work ability, and high employability throughout their career.

Studies suggest that job demands and job resources may influence sustainable employability. For instance, various interventions designed to help employees manage their job demands and job resources (e.g., through job crafting or stimulating work engagement) have been favourably associated potential causes and indicators of sustainable employability, including vitality, work pressure, and employability [28, 35–37]. Given that teleworking may be linked to job demands such as work pressure, and job resources such as social support, workplace communication, and role clarity, it is plausible to assume that telework may be associated with sustainable employability through such job demands and job resources. Below, we outline the different pathways through which job demands and job resources may connect telework and sustainable employability.

### 1.3 Telework and sustainable employability: The role of work pressure

According to the JD-R model [14, 15], an increase in work pressure, defined by Roe and Zijlstra [38] as “a subjective state of tension associated with the current and/or anticipated execution of work tasks” (p. 1), could lead to work stress and burnout when employees possess insufficient job resources to mitigate the added pressure. Therefore, work pressure has a negative effect on employees’ work ability. Furthermore, such an increase in work stress is negatively related to work engagement [18], of which vitality is an integral part [10]. It is also likely that employees under high work pressure do not have enough time to invest in their personal development on top of their regular tasks. This personal development and the sharing of knowledge are known to improve employability [10]. It is thus plausible that higher work pressure is related to lower levels of vitality, work ability, and employability (i.e., sustainable employability).

Jamal and colleagues [39] noted that employees who teleworked during the COVID-19 pandemic reported higher work pressure, task interdependence, professional isolation, and family interference, leading to higher exhaustion and stress. A later study by Bekkers [40] identified new stressors caused by teleworking that could be related to work pressure, such as digital aggression and the pressure to respond to e-mails quickly (tele-pressure). Furthermore, employees’ work-life balance could be negatively linked to teleworking: when non-work-related activities detract from one’s working hours, this could increase one’s experienced work pressure [41]. Based on the above, the first group of hypotheses was formulated:

**H1a**: *Teleworking is positively related to work pressure, such that employees who conduct more telework experience higher work pressure.*

**H1b**: *Work pressure is negatively related to the three elements of sustainable employability, i.e., (i) employability, (ii) work ability, and (iii) vitality, such that employees who experience higher work pressure report lower employability, work ability, and vitality.*

**H1c:** *The relationships between teleworking and respectively (i) employability, (ii) work ability, and (iii) vitality are partially mediated by work pressure.*

### 1.4 Telework and sustainable employability: The role of social support

The job resource social support has been defined in many ways, encompassing support through social ties, a coping asset, being cared for, and being appreciated and valued [42, 43]. In the current study, we focus specifically on social support in the workplace. Brouwers and colleagues [44] showed that a lack of social support from coworkers was related to a larger chance of burnout complaints, which indicates a decreased work ability [10]. Conversely, the presence of social support can reduce the chance of these complaints [45]. It is worth noting that burnout complaints typically include an absence of motivation, the opposite of which is one of the characteristics of vitality [46].

The importance of social support is further underlined by Jolly and associates [47], who explain how social support contributes to better relationships, more positive affective reactions, and better individual work accomplishments. These improved relationships and positive support are also considered predictors of employability [9]. Social support thus creates a context in which employees are more likely to develop and remain motivated, improving their employability and vitality [10, 48, 49].

A teleworking enquiry in the Netherlands [50] showed that 75% of employees who engaged in teleworking felt that they missed their colleagues, providing further evidence of the relationship between teleworking and social support. Prior to the COVID-19 pandemic, Sardeshmukh and colleagues [19] analysed the relationships between teleworking and several job demands (e.g., time pressure, role ambiguity, role conflict) and job resources (e.g., autonomy, feedback, social support), and found, among others, a negative relationship between teleworking and social support. Based on the above, the second group of hypotheses was formulated:

**H2a**: *Teleworking is negatively associated with social support, such that employees who conduct more telework experience lower social support.*

**H2b**: *Social support is positively associated with the three elements of sustainable employability, i.e., (i) employability, (ii) work ability, and (iii) vitality, such that employees who experience higher social support also report higher employability, work ability, and vitality.*

**H2c:** *The relationships between teleworking and respectively (i) employability, (ii) work ability, and (iii) vitality are partially mediated by social support.*

### 1.5 Telework and sustainable employability: The role of workplace communication

The quality of communication between employees and supervisors is of great importance in daily work situations, as it contributes to employability through learning and personal development [34]. Indeed, workplace communication is essential for knowledge sharing, providing feedback, and motivating employees [51]. Furthermore, the extent to which employees experience communication to be positive contributes to how open they are to receiving feedback, which is essential to foster learning [52]. The motivational aspect of good communication may contribute to the mental state and thus to the vitality of employees [31]. In addition, learning from supervisors and receiving clear feedback are of importance to keep working in a healthy manner [10], which suggests a relationship with work ability as well. Meaningful workplace communication allows employees and supervisors to resolve problems together when they arise [53], and may prevent problems from accumulating and causing stress [46], which benefits employee health and thus their work ability [33].

The previously mentioned teleworking enquiry [50] also demonstrated that 44% of employees experienced meeting over the phone as difficult, indicating clear difficulties in workplace communication while teleworking. Additionally, a negative relationship between teleworking and amount of feedback was found [19]. Analogously, Watson-Manheim and colleagues [54] investigated need differences in workplace communication during telework, and found, among other things, that telecommuters do need occasional (online) face-to-face communication and/or phone calls to support their performance, possibly to make up for the lack of casual workplace conversation. Moreover, they found differing preferences in the scheduling of phone calls between telecommuters and non-telecommuters, with the former preferring scheduled conversations and the latter preferring scheduled conversations, indicating differing workplace communication needs between these groups of employees. Based on the theoretical outline given above, the third group of hypotheses was formed:

**H3a**: *Teleworking is negatively associated with the prevalence of and satisfaction with workplace communication, such that employees who conduct more telework experience less satisfactory workplace communication.*

**H3b**: *The prevalence of and satisfaction with workplace communication is positively associated with the three elements of sustainable employability, i.e., (i) employability, (ii) work ability, and (iii) vitality, such that employees who experience more satisfactory workplace communication also report higher employability, work ability, and vitality.*

**H3c:** *The relationships between teleworking and respectively (i) employability, (ii) work ability, and (iii) vitality are partially mediated by the prevalence of and satisfaction with workplace communication.*

### 1.6 Telework and sustainable employability: The role of role clarity

Role clarity encompasses the clarity of responsibilities, work content, and work processes, regarding an employee’s own position and the positions of their colleagues [55]. In the context of the JD-R model, role ambiguity (i.e., low role clarity) has been found to cause stress, and to positively relate to burnout [46], and as such to affect employee health and work ability. Additionally, role ambiguity negatively relates to work engagement [46], of which vitality is an integral part [56]. When there is little work engagement, the odds of employees increasing their efforts to further develop their skills and knowledge are smaller [57], which could in turn impact their employability [10]. The previously mentioned telework study by Sardeshmukh and colleagues [19] showed a positive relationship between teleworking and role ambiguity. Moreover, changes in the way work is done while teleworking during COVID-19, for example through the use of new technologies and through differing workloads, can make employees experience role ambiguity as they feel that their responsibilities are unclear [58]. In addition, they might experience difficulties in communication as teleworking can further increase role ambiguity [59]. Given the likely relationships between telework, role clarity and the elements of sustainable employability, the fourth group of hypotheses was formulated:

**H4a**: *Teleworking is negatively associated with role clarity, such that employees who conduct more telework experience lower role clarity.*

**H4b**: *Role clarity is positively associated with the three elements of sustainable employability, i.e., (i) employability, (ii) work ability, and (iii) vitality, such that employees who experience higher role clarity also report higher employability, work ability, and vitality.*

**H4c:** *The relationships between teleworking and respectively (i) employability, (ii) work ability, and (iii) vitality are partially mediated by role clarity.*

## 2 Methods

### 2.1 Participants and procedure

This study was conducted by means of a digital survey among the personnel base of a government agency in the Netherlands, which consisted of approximately 1,600 employees. Data collection took place in April and May 2021, while Dutch COVID-19 restrictions were in place and most employees were required to work from home. All employees were informed about the study and were invited to participate in the survey via email. Employees were also given the option to withdraw from the study at any point in time. Voluntary participation led to a sample size of approximately 35% of the total personnel base. Responses from participants who did not complete the questionnaire were excluded from the analyses. The final sample thus consisted of data from 552 participants (46.3% male, 53.5% female, 0.2% ‘other’). The average age of participants was 49.65 years (*SD* = 10.86, range = 18–66), and 69.7% completed an education at a university or at a university of applied sciences. The characteristics of the final sample were representative of the total personnel base of the organisation with regards to gender, age, and educational level.

As the survey was distributed to the entire personnel base of our target organisation, we note that the average age of participants in our final sample is relatively high. We believe that this relatively high average age calls attention to the fact that the working population in many countries across the world is ageing, with the Netherlands being no exception (in 2021, 30% of the Dutch working population was already over-fifty [8]). This ageing workforce will ultimately contribute to an increasing number of retired people and a decline in the working population. As such, it is of utmost importance to gain further insight into how we can protect and further enhance the sustainable employability of the workforce throughout the lifespan.

### 2.2 Measures

#### 2.2.1 Job demands and job resources

Following a qualitative preliminary investigation into the specific job demands and job resources that were experienced as significant within the studied organisation, using existing data from employee satisfaction surveys and nine semi-structured interviews with employees in the HR department (three interviews) and a number of employees in various different functions and departments of the organisation (six interviews), the job demand ‘work pressure’ and the job resources ‘social support’, ‘workplace communication’ and ‘role clarity’ were selected as the main job demands and job resources to focus on in the current study:

*Work pressure*. The job demand ‘work pressure’ was measured using three items (Cronbach’s *α*=.86) from the TNO Dutch Work Conditions Questionnaire [60]. For these items, a 4-point Likert scale was used consisting of the following answering categories: “*never*” (1), “*sometimes*” (2), “*often*” (3), and “*always*” (4). A translation of an example question that was used is: “*Do you have to work very fast?*”. Higher mean scores on these three items reflected higher work pressure.

*Social support.* The job resource ‘social support’ was measured using four items (Cronbach’s *α*=.72) from the TNO Dutch Work Conditions Questionnaire [60]. For these items, a 4-point Likert scale was used consisting of the following answering categories: “*never*” (1), “*sometimes*” (2), “*often*” (3), “*always*” (4), and “*not applicable*” (all respondents who scored the latter were excluded from all further analyses). A translated example of a used statement is “*My employer notices the well-being of employees.*” Higher mean scores on these items indicated higher social support.

*Workplace communication.* The job resource ‘workplace communication’ was measured using four items that were all scored on a 5-point Likert scale from the Perceived Leadership Communication Questionnaire [51] that were translated to Dutch (Cronbach’s *α*= .73). The specific aspects of workplace communication that were measured comprised the prevalence of and satisfaction with interpersonal communication, both between employees and their supervisors, and between employees and their direct colleagues. An example of a statement that was used is: “*Especially when problems arise, we talk to one another even more intensively in order to solve the problems*”. Answering categories were: “*Completely disagree*” (1), “*Disagree*” (2), “*Neutral*” (3), “*Agree*” (4), and “*Completely agree*” (5). Higher mean scores on these items suggested higher workplace communication satisfaction.

*Role clarity*. The job resource ‘role clarity’ was measured using four items that were scored on a 4-point rating scale from Rizzo and colleagues [61], which were translated into Dutch (Cronbach’s *α*=.80). Participants were asked to which extent they agreed with a statement. Examples of statements were: “*I know what my responsibilities are*” and “*I know exactly what is expected of me*”. Answering categories were “*never*” (1), “*sometimes*” (2), “*often*” (3), “*always*” (4), and “*not applicable*” (respondents scoring this answer were excluded from all further analyses). Higher mean scores on these items indicated higher role clarity.

#### 2.2.2 Sustainable employability

*Vitality.* Vitality was measured with questions from the Utrecht work engagement scale (Utrechtse Bevlogenheid Schaal [62]), consisting of six items that were scored on a 5-point Likert scale (Cronbach’s *α*=.77). Validated translations of example statements were: “*I am very energetic at work*” and “*When I work I feel fit and strong*”. Answering categories were: “*Not at all*” (1), “*Rarely*” (2), “*Sometimes*” (3), “*Often*” (4), and “*Very often*” (5). Higher mean scores on these items suggested higher overall vitality.

*Work ability*. Work ability was measured by means of the organisation’s sick leave data for the year 2020. Both the *duration* and *frequency of sick leave* were used as indicators of work ability. Previous research by Van Vuuren and colleagues [63] argued for the use of sick leave data as a work ability measure, referring to the original work ability model by Ilmarinen and associates [32] which considered good health as the most important indicator of work ability.

*Employability*. Employability was measured using the short-form version [64] of the employability questionnaire created by Van der Heijde and Van der Heijden [33], consisting of 22 validated items, each rated on a 6-point scale (Cronbach’s *α*=.88). The answering options varied between items. A translated example of a statement was used is: “*My work and private life are*  ...   *balanced.*” Answering options for this statement were “*Not at all*” (1), “*Scarcely*” (2), “*Not to all that great degree*” (3), “*To a fairly great degree*” (4), “*To a great degree*” (5) and “*To a considerable degree*” (6). Higher mean scores on these items reflected higher employability.

*Teleworking.* Teleworking was measured as the percentage of time employees work from home. To this end, they were asked to indicate the average number of hours they work from home on a weekly basis. This number was calculated as a percentage of the total number of working hours. A higher percentage reflected a higher propensity to work from home.

*Working hours.* The average number of hours worked per week was added as a control variable in the analysis, as this may relate to the percentage of time one is teleworking. Moreover, the number of working hours can also relate to the experienced work pressure because there might be less time to complete one’s tasks. The variable working hours was measured by asking participants to indicate their average total number of working hours per week.

### 2.3 Data analysis

**Fig.1:**
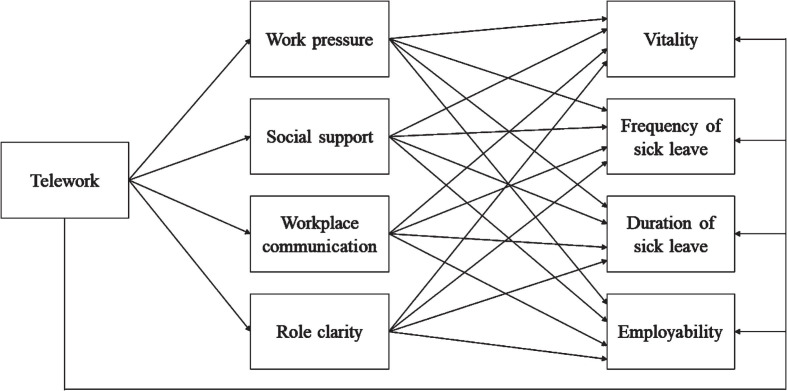
Hypothesised path model of the relationship between telework, job demands (work pressure), job resources (social support, workplace communication, and role clarity), and sustainable employability outcomes (vitality, work ability, and employability). Participants’ work ability was measured through their frequency and duration of sick leave.

To investigate the relationship between teleworking, job demands (work pressure), job resources (social support, workplace communication, role clarity), and sustainable employability outcomes (vitality, work ability, employability), we specified and fitted a path model of our study variables in R. Based on the hypothesised relationship between our study variables, teleworking was modelled to be directly related to each of the job demands and resources included in the study, as well as to the indicators of sustainable employability (Fig. 1). In addition to these direct effect paths, we specified indirect effect paths for the effect of teleworking on each of the sustainable employability outcomes, through each of the job demands and resources. Both job demands and job resources were thus positioned as mediators of the effect of telework on sustainability outcomes in our path model, in line with prior studies on telework and factors relating to sustainable employability (e.g., Crawford [65], Jamal et al. [39], Lopes et al. [66], Sardeshmukh et al. [19]).

We used full information maximum likelihood estimation with bootstrapped confidence intervals to fit our path model. To assess goodness-of-fit, we utilized the recommended practical guidelines of RMSEA <  = .06, NNFI > =. 95, CFI > =. 95, and SRMR < =. 08 [67]. When the model did not adequately fit our data, we consulted modification indices to exclude or include additional effect paths, as long as these paths made theoretical sense. Finally, the interpretation of each effect path was done with a standard criterion of *α*= 0.05.

## 3 Results

### 3.1 Descriptive statistics

Descriptive statistics and correlation coefficients between all model variables are presented in Table 1.

**Table 1:** Means, standard deviations and correlations of variables

	*M*	*SD*	1	2	3	4	5	6	7	8	9
1. Work Pressure	2.61	0.63
2. Social Support	3.39	0.49	–.01
3. Workplace Communication	3.63	0.62	–.01	.59^ **^
4. Role Clarity	3.11	0.54	–.02	.33^ **^	.37^ **^
5. Vitality	3.68	0.54	.16^ **^	.26^ **^	.28^ **^	.32^ **^
6. Sick leave duration	15.33	51.85	.01	–.12^ **^	–.11^ *^	–.04	–.17^ **^
7. Sick leave frequency	0.55	0.99	–.01	–.12^ **^	–.16^ **^	.02	–.16^ **^	.27^ **^
8. Employability	4.21	0.48	.12^ **^	.29^ **^	.31^ **^	.40^ **^	.55^ **^	–.20^ **^	–.13^ **^
9. Teleworking	0.83	0.76	.04	.01	–.01	–.12^ **^	–.05	–.05	–.07	–.03
10. Working hours (total)	34.14	6.96	.33^ **^	.02	.02	–.02	.13^ **^	–.19^ **^	–.02	.19^ **^	–.17^ **^

*n*: between 473 and 557, ^ *^p <.05; ^ **^p <.01.

### 3.2 Assessment of model fit

To investigate the relationships between teleworking, job demands, job resources, and sustainable employability outcomes, we first fitted our hypothesised path model (Fig. 1). Fit indices for this hypothesised model suggested that the model poorly fitted our data, *χ*^2^(7) = 32.536, *p* <  .001; RMSEA = .090, NNFI = .765, CFI,.963, SRMR = .037. We report the estimated direct and indirect effects for this model in Appendix 1 (Table A1). To improve model fit, we examined modification indices for this initial model to determine whether additional, theoretically plausible paths could be included. Based on the outcomes, we added working hours as a predictor for employability and duration of sick leave. We report all modification indices that were obtained for the initial model in Appendix 2 (Table A2). The improved model adequately fitted our data, *χ*^2^(5) = 8.747, *p* <  .001; RMSEA = .041, NNFI = .952, CFI,.995, SRMR = .019. The model accounted for a relatively modest proportion of variance in work pressure (*R^2^* = 12.8%), social support (*R^2^* = .1%), workplace communication (*R^2^* = .01%), role clarity (*R^2^* = 1.2%), vitality (*R^2^* = 14.5%), duration of sick leave (*R^2^* = 5.4%), frequency of sick leave (*R^2^* = 3.5%), and employability (*R^2^* = 25.0%). Table 2 provides an overview of all direct and indirect effects that were estimated in the improved path model.

**Table 2:** Standardized estimates of direct and indirect effects in the relationship between telework, job characteristics, and sustainable employability outcomes

Path	*β*	SE	95% Confidence Interval
Direct effects
Percentage telework⪢work pressure	.13	.05	[.04,.22]^ *^
Percentage telework⪢social support	.03	.05	[–.07,.13]
Percentage telework⪢communication	.01	.05	[–.09,.11]
Percentage telework⪢role clarity	–.10	.05	[–.19, –.01]^ *^
Percentage telework⪢vitality	–.06	.05	[–.16,.03]
Percentage telework⪢duration of sick leave	–.10	.06	[–.22,.03]
Percentage telework⪢frequency of sick leave	–.14	.06	[–.24, –.03]^ *^
Percentage telework⪢employability	.03	.05	[–.06,.11]
Work pressure⪢vitality	.15	.05	[.05,.24]^ *^
Work pressure⪢duration of sick leave	.08	.07	[–.05,.21]
Work pressure⪢frequency of sick leave	.00	.05	[–.01,.11]
Work pressure⪢employability	.06	.05	[–.03,.15]
Social support⪢vitality	.09	.06	[–.02,.19]
Social support⪢duration of sick leave	–.10	.06	[–.22,.01]
Social support⪢frequency of sick leave	–.05	.05	[–.15,.05]
Social support⪢employability	.09	.05	[–.01,.18]
Communication⪢vitality	.13	.05	[.04,.23]^ *^
Communication⪢duration of sick leave	–.05	.08	[–.20,.10]
Communication⪢frequency of sick leave	–.10	.06	[–.22,.03]
Communication⪢employability	.13	.05	[.03,.26]^ *^
Role clarity⪢vitality	.22	.05	[.13,.31]^ *^
Role clarity⪢duration of sick leave	.02	.04	[–.06,.09]
Role clarity⪢frequency of sick leave	.06	.05	[–.04,.16]
Role clarity⪢employability	.37	.04	[.29,.46]^ *^
Working hours⪢work pressure	.33	.05	[.24,.42]^ *^
Working hours⪢social support	.01	.05	[–.09,.11]
Working hours⪢communication	.01	.05	[–.09,.11]
Working hours⪢role clarity	–.05	.05	[–.13,.04]
Working hours⪢duration of sick leave	–.18	.08	[–.33, –.02]^ *^
Working hours⪢employability	.14	.04	[.06,.22]^ *^
Indirect effects
Percentage telework⪢work pressure⪢vitality	.02	.01	[.00,.04]^ *^
Percentage telework⪢social support⪢vitality	.00	.01	[–.01,.01]
Percentage telework⪢communication⪢vitality	.00	.01	[–.01,.01]
Percentage telework⪢role clarity⪢vitality	–.02	.01	[–.04,.00]
Percentage telework⪢work pressure⪢duration of sick leave	.01	.01	[–.01,.03]
Percentage telework⪢social support⪢duration of sick leave	–.01	.01	[–.03,.00]
Percentage telework⪢communication⪢duration of sick leave	–.01	.01	[–.03,.02]
Percentage telework⪢role clarity⪢duration of sick leave	.00	.01	[–.01,.01]
Percentage telework⪢work pressure⪢frequency of sick leave	.00	.01	[–.01,.01]
Percentage telework⪢social support⪢frequency of sick leave	–.01	.01	[–.02,.01]
Percentage telework⪢communication⪢frequency of sick leave	–.01	.01	[–.03,.01]
Percentage telework⪢role clarity⪢frequency of sick leave	.01	.01	[–.01,.02]
Percentage telework⪢work pressure⪢employability	.01	.01	[–.01,.02]
Percentage telework⪢social support⪢employability	.01	.01	[–.00,.03]
Percentage telework⪢communication⪢employability	.02	.01	[–.00,.03]
Percentage telework⪢role clarity⪢employability	.05	.02	[.01,.08]^ *^

### 3.3 Relationships between telework, job demands, and job resources

With regards to the hypothesised relationships between telework, job demands (work pressure), and job resources (social support, workplace communication, role clarity), the improved path model suggested that employees who spent a larger percentage of their time teleworking experienced higher work pressure (Hypothesis 1a) and less role clarity (hypothesis 4a). Conversely, the improved model provided little support for the association between telework percentage and social support (Hypothesis 2a) and the association between telework percentage and workplace communication (Hypothesis 3a). This implies that a higher percentage of time spent teleworking had little-to-no relationship with employees’ social support and workplace communication.

### 3.4 Relationships between job demands, job resources, and sustainable employability

With respect to the hypothesised relationships between job demands, job resources, and sustainable employability outcomes (vitality, duration of sick leave, frequency of sick leave, employability), the improved path model suggested that employees who reported higher work pressure also experienced higher vitality. This effect is opposite of what we hypothesized for the relationship between work pressure and vitality (Hypothesis 1b, part iii). No significant associations were found between work pressure and the remaining sustainable employability outcomes. Thus, the improved model provides little support for the hypothesized relationships between job demands and the three dimensions of sustainable employability, namely employability, work ability, and vitality (Hypothesis 1b).

Regarding job resources, the improved path model suggested that employees who reported greater prevalence of and satisfaction with interpersonal workplace communication experienced higher employability (Hypothesis 3b, part i) and higher vitality (Hypothesis 3b, part iii). Similarly, employees who reported higher role clarity also experienced higher employability (Hypothesis 4b, part i) and higher vitality (Hypothesis 4b, part iii). No significant association was found between workplace communication, role clarity, and both the duration and frequency of sick leave. The improved path model also found no significant associations between social support and sustainable employability, herewith not supporting the entirety of Hypothesis 2b.

### 3.5 Indirect effects of job demands and job resources

Finally, with regards to the hypothesised mediating role of job demands and job resources in the relationship between telework and sustainable employability, the estimated effects in the path model suggest that the association between telework percentage and vitality may be mediated by the job demand work pressure (Hypothesis 1c, part iii). Similarly, the association between telework percentage and employability may be mediated by the job resource role clarity (Hypothesis 4c, part i), No support was found for the remaining parts of Hypothesis 1c and Hypothesis 4c, and all parts of Hypotheses 2c and 3c.

## 4 Discussion

The COVID-19 pandemic has motivated a rapid rise in the adoption of telework, as organisations and employees wrestled with relevant health – and safety regulations at the time. The current study took place in the context of mass telework due to COVID-19 restrictions. Rather than teleworking for the now increasingly common two or three days per week, most employees were often obligated to telework their entire workweek. As organisations are investing less in office spaces, and some large organisations choose to go without office space altogether, the present study aimed to further our understanding of the impact of such mass telework on employees. To do so, the present study examined the relationship between telework, the job demand ‘work pressure’, the job resources ‘social support’, ‘workplace communication’, and ‘role clarity’, and their subsequent relationships with sustainable employability (operationalized as vitality, work ability, and employability). A path model of the hypothesised relationship between the study variables suggested that employees who spent a larger percentage of their time teleworking reported higher work pressure and less role clarity. Employees who experienced more job demands in the form of work pressure reported higher vitality. Further, there was a positive association between the job resources ‘workplace communication’ and ‘role clarity’ on the one hand, and sustainable employability outcomes ‘vitality’ and ‘employability’ on the other. No significant association was found between social support and sustainable employability despite prior studies suggesting a strong correlational relationship between these variables. Finally, the results suggested that the relationship between teleworking and vitality may be mediated by work pressure, while the relationship between teleworking and employability may be mediated by role clarity. However, caution should be taken in interpreting these mediating effects given the cross-sectional nature of the study data.

Collectively, several associations uncovered by our study are surprising from the perspective of the JD-R model [14]. For instance, being a job demand, we expected work pressure to have a negative relationship with teleworking employees’ sustainable employability. On the contrary, our results show a clear positive relationship between work pressure and sustainable employability, which begs the question whether work pressure, as measured in the current study, truly functions as a stressor for employees. A likely explanation is that employees feel the need to be challenged at work: insufficiently challenging work could lead to less motivation and work-related boredom [68]. In other words, work pressure in a healthy quantity can improve mental state, whereas in excessive quantities it can negatively affect mental health [46]. Given that job demands are not considered stressful unless they exceed a relative threshold [14, 18], it is plausible to assume that the average reported work pressure in this study (2.61 out of 5) may not have exceeded this threshold, and was thus considered motivating by the participants in our study.

Next, the results suggested no statistically significant association between teleworking, the job resource ‘social support’, and sustainable employability outcomes. Similarly, there was little evidence for relationships between teleworking and workplace communication. The organisation in which this study took place made use of many online communication tools (e.g., Microsoft teams, Trello), which may have been effective in maintaining sufficient communication throughout the pandemic. However, our results do suggest a significant positive association between the job resource ‘workplace communication’ and the sustainable employability outcomes ‘vitality’ and ‘employability’. Taken together, these results run contrary to the findings by Sardeshmukh and colleagues [19], who presented a negative relationship between teleworking and respectively social support and communication.

Finally, the results indicated that teleworking was negatively associated with role clarity. In addition, the results showed that role clarity was positively and significantly associated with vitality and employability, but not with work ability. Interestingly, the relationship between teleworking and employability may be mediated by role clarity. However, as with the potential mediating role of work pressure in the relationship between teleworking and vitality, we opted to interpret the indirect effect of role clarity with caution given the cross-sectional nature of our data. Taken together, the findings on role clarity are in accordance with the theory that teleworking relates to role ambiguity [19]. The results support the notion that role clarity (regardless of teleworking) is associated with vitality and employability [10]. According to Bakker and Schaufeli [57], changes in role clarity may influence the extent to which employees are engaged with their work, of which vitality is an integral part [56]. In turn, this affects the extent to which employees engage in personal development, which ultimately affects their employability. While it was not possible to examine this chain of events within the present study, the current results provide valuable insight into the potential associations between teleworking, role clarity, and employability.

It is also worth mentioning that contrary to our assumption that the presence of job resources would reduce the chance of sick leave, almost none of the job demands and job resources in the current study appeared to be related to sick leave, and thus work ability. An exception to this is that a higher prevalence and satisfaction with workplace communication was related to a lower frequency of sick leave. This finding is in accordance with the principle that supervisors can leverage effective communication to prevent employee stress levels from rising to a threshold that may cause sick leave [32, 46]. The lack of statistically significant associations between job demands and job resources, on the one hand, and work ability on the other hand, may also be explained by the high infection rates of COVID-19, which led to an increase in both short- and long-term sick leave during the pandemic [69]. Thus, the proportion of sick leave that could be attributed to the presence of job demands and job resources was relatively smaller in the pandemic situation. Moreover, the increase in teleworking may have caused presenteeism, as employees either continue to work from home despite being ill, or take the day off work without feeling the need to report being ill [70]. It is more plausible that this would occur in the case of complaints that relate to job demands and job resources than with non-psychological complaints, due to the stigma on psychological complaints [71].

We have tried to answer the question of whether teleworking in the context of COVID-19 relates to certain job demands and job resources that could ultimately relate to sustainable employability. We have found that teleworking for the largest part does not directly relate to sustainable employability, but that it does negatively relate to the frequency of sick leave. This relationship may however be caused by presenteeism. Teleworking does seem to relate to both work pressure and role clarity, each of which in turn relates to factors of sustainable employability. An indirect relationship between teleworking and sustainable employability may thus exist but should be interpreted with caution given the cross-sectional nature of this study. From these findings no major risks of mass teleworking thus become apparent for the sustainable employability of personnel. However, the higher work pressure and lower role clarity that we found to be associated with teleworking may affect the functioning and wellbeing of employees throughout their teleworking careers.

### 4.1 Limitations and recommendations

There are several limitations to this study that are important to discuss. First, most of the data used for the analyses were gathered through self-reports, with the sick-leave-related (work ability) variables being the exception. While self-reported measures are often more vulnerable to common-method biases, (e.g., social desirability, positive and negative affectivity) the use of self-report measures in the current study is arguably justifiable, as our data concern the anonymised experiences of employees themselves. However, the perspective of colleagues or supervisors for example could have provided a more complete representation of the relationship between teleworking, job demands, and job resources, and sustainable employability.

Second, the survey was not mandatory, and thus it is possible that a response bias exists. Participants could moreover choose to opt out of sharing their sick leave data. It is thus possible that employees with longer or more frequent sick leaves were less inclined to share their data, causing this group to be underrepresented in the sample. Contrarily, it is also possible that employees for whom sick leave, teleworking, job demands, and job resources do not play a large role did not consider the study personally relevant, and for that reason did not participate. Despite this, the demographic characteristics (i.e., age, gender) of our sample provided a reasonably accurate representation of the organisation in which the study was conducted. Furthermore, the total sick leave data from the participating organisation is comparable to the sample data in terms of both sick leave duration (*M* = 19.25, *SD* = 57.15) and frequency of sick leave (*M* = 0.62, *SD* = 0.98), thus making it likely that both employees with higher sick leave scores and employees with lower sick leave scores consented to the use of their sick leave data.

Lastly, due to the lack of longitudinal data or an experimental setting, causal relationships cannot be confirmed by this study, but merely suggested [72]. Similarly, while the decision to analyse indirect relationships was based on prior studies on teleworking and factors relating to sustainable employability (e.g., Crawford [65], Jamal et al. [39], Lopes et al. [66], Sardeshmukh et al. [19]), we have chosen to interpret the indirect effects in our model very cautiously given the cross-sectional nature of our data, as robust tests of mediation require a temporal order between predictors, mediators, and outcomes, that are challenging to identify in cross-sectional data [73]. Thus, to verify the potential mediating roles of work pressure and role clarity in the relationship between teleworking and sustainable employability, we recommend future studies to adopt a longitudinal design. However, it is important to note that there is not yet any certainty about the time frame in which job demands and resources may impact sustainable employability. Dormann and Griffin [74] for example found that oftentimes shorter time lags than those commonly used in research are justifiable (cf. Lesener et al. [75]), and argue for more “shortitudinal” studies. In other words, longer time lags may not be as effective as shorter time lags at obtaining more comprehensive portraits of the relationship between job demands and job resources, on the one hand, and sustainable employability, on the other hand, as job demands such as work pressure can be present in peaks and their impact could be quite direct, for example in the case of acute stress [76]. As such, the mediation hypotheses in the current study, though not tested over a span of time, could still give a preliminary indication of possible pathways that may exist, especially given their theoretical underpinnings. Future longitudinal studies investigating the interplay between teleworking, job demands, and job resources, and sustainable employability should thus be considerate of the time frames over which the studied job demands and job resources are likely to have an effect.

The cross-sectional nature of the current study leaves room for several alternative explanations for the results that were found. It could be that more vital, motivated employees have a more positive attitude, and are more likely to notice positive work characteristics due to selective perception [77]. The presence of job demands and job resources could moreover have influenced the choices that employees make regarding the amount of time they spend teleworking to the extent they are able to decide about this themselves. Further research could investigate a causal relationship through collecting and analysing longitudinal data regarding teleworking, job demands and job resources, and sustainable employability.

It should furthermore be noted that the current study investigated specific job demands and job resources. Although these specific choices are based on a thorough literature study and a qualitative pre-study, some possibly relevant job demands and job resources (for other organisations) could have been overlooked. We therefore recommend that in future studies the presence of other job demands and resources in the context of teleworking should be studied, as well as their relationship to sustainable employability.

Crucially, the data collection procedure for the current study was conducted at the height of the COVID-19 pandemic, when telework was mandated for most organisations according to relevant health – and safety regulations. As such, the percentage of teleworking sampled in this study may be over-representative of the actual telework percentage in most organisations at the present time. Consequently, the outcomes regarding the relationships under study may apply only to organisations that put a higher emphasis on telework compared to others (e.g., remote first companies, etc.). When cross-validating our outcomes using a current sample and comparing these with the results of this empirical work, it might be possible that the statistical significance of some of the relationships examined in our path model may either be over- or underestimated. Future studies could look into the generalizability of our outcomes across occupational sectors and countries.

### 4.2 Practical implications and theoretical contributions

The current study provides insights for the possibilities for teleworking in the future, and the job demands and job resources that can be used to manage teleworking in a manner that promotes sustainable employability. While no negative relationships were found between work pressure and sustainable employability, the results do show that teleworking relates to higher work pressure. This warrants an alertness to work pressure in the context of teleworking; organisations should take caution that teleworking does not cause the level of work pressure to become a stressor, for example by ensuring that the work pressure for employees who continue to work from home is adequately monitored. Supervisors could play a key role in this by checking in with their employees and enquiring about their workload on a regular basis. Immediate action can then be taken when the work pressure exceeds acceptable levels.

Tentatively, the results highlight the importance of role clarity in the relationship between teleworking and sustainable employability, thus providing a point of action for organisations in which teleworking remains common. As teleworking may negatively impact role clarity, and role clarity was found to be positively associated with sustainable employability outcomes (vitality and employability), it may be useful for organisations to promote role clarity to ensure that teleworking employees may remain sustainably employable. One way to promote role clarity for employees would be to make use of so-called ‘balanced scorecards’: employee, supported by their supervisor, drafts an overview of personal goals, tasks, development needs, and measurements for success in their function, herewith creating clarity about needs, tasks, and expectations both on the side of the employee and the supervisor [78]. Another way of promoting role clarity, especially for new employees, is to ensure a thorough onboarding process for new employees, and paying extra attention to how this can be done in the context of teleworking [79].

In the current study, we found a direct positive relationship between teleworking and work pressure, and direct negative relationships between teleworking and role clarity and the frequency of sick leave, respectively. We also found positive direct relationships between work pressure, workplace communication, and role clarity on the one hand, and indicators of sustainable employability on the other hand. Lastly, although this issue should be interpreted with caution, given the cross-sectional nature of the data, we found positive effects of teleworking on indicators of sustainable employability through work pressure and role clarity, being the mediators. We did not find a negative relationship between teleworking and sustainable employability in this empirical study.

## 5. Conclusion

The present study offers a positive perspective on the future of telework, which is especially relevant given that many employees indicate a preference for (partially) teleworking [80], and as many organisations continue to allow or even encourage teleworking [6]. While our results reflect few expected relationships between job demands and job resources on the one hand, and sick leave on the other hand, the present study serves as a reminder for organisations to be aware of the manner in which teleworking could potentially stimulate presenteeism, and to take notice that employees may not call in sick while actually being ill as they are less visible in the workplace.

## Appendix 1

**Table A1:** Standardized estimates of direct and indirect effects in the initial model of the relationship between telework, job characteristics, and sustainable employability outcomes

Path	*β*	SE	95% Confidence Interval
Direct effects
Percentage telework⪢work pressure	.13	.05	[.03,.23]^ *^
Percentage telework⪢social support	.03	.05	[–.06,.12]
Percentage telework⪢communication	.01	.05	[–.10,.11]
Percentage telework⪢role clarity	–.10	.05	[–.19, –.01]^ *^
Percentage telework⪢vitality	–.06	.05	[–.15,.03]
Percentage telework⪢duration of sick leave	–.09	.06	[–.22,.03]
Percentage telework⪢frequency of sick leave	–.14	.06	[–.25, –.02]^ *^
Percentage telework⪢employability	.02	.04	[–.06,.11]
Work pressure⪢vitality	.15	.05	[.05,.24]^ *^
Work pressure⪢duration of sick leave	.02	.06	[–.10,.15]
Work pressure⪢frequency of sick leave	.00	.05	[–.10,.11]
Work pressure⪢employability	.09	.05	[–.00,.18]
Social support⪢vitality	.09	.06	[–.02,.20]
Social support⪢duration of sick leave	–.11	.06	[–.23,.02]
Social support⪢frequency of sick leave	–.05	.05	[–.15,.06]
Social support⪢employability	.09	.05	[–.01,.18]
Communication⪢vitality	.13	.05	[.04,.23]^ *^
Communication⪢duration of sick leave	–.05	.08	[–.21,.11]
Communication⪢frequency of sick leave	–.10	.06	[–.22,.03]
Communication⪢employability	.13	.05	[.03,.23]^ *^
Role clarity⪢vitality	.22	.05	[.13,.32]^ *^
Role clarity⪢duration of sick leave	.03	.04	[–.05,.11]
Role clarity⪢frequency of sick leave	.06	.05	[–.04,.16]
Role clarity⪢employability	.36	.04	[.28,.45]^ *^
Working hours⪢work pressure	.33	.05	[.24,.42]^ *^
Working hours⪢social support	.01	.05	[–.09,.11]
Working hours⪢communication	.01	.05	[–.09,.11]
Working hours⪢role clarity	–.05	.05	[–.14,.05]
Indirect effects
Percentage telework⪢work pressure⪢vitality	.02	.01	[.00,.04]^ *^
Percentage telework⪢social support⪢vitality	.00	.01	[–.01,.01]
Percentage telework⪢communication⪢vitality	.00	.01	[–.01,.02]
Percentage telework⪢role clarity⪢vitality	–.02	.01	[–.04,.00]^ *^
Percentage telework⪢work pressure⪢duration of sick leave	.00	.01	[–.01,.02]
Percentage telework⪢social support⪢duration of sick leave	–.01	.01	[–.03,.00]
Percentage telework⪢communication⪢duration of sick leave	–.01	.01	[–.03,.02]
Percentage telework⪢role clarity⪢duration of sick leave	.00	.01	[–.01,.01]
Percentage telework⪢work pressure⪢frequency of sick leave	.00	.01	[–.01,.01]
Percentage telework⪢social support⪢frequency of sick leave	–.01	.01	[–.02,.01]
Percentage telework⪢communication⪢frequency of sick leave	–.01	.01	[–.03,.01]
Percentage telework⪢role clarity⪢frequency of sick leave	.01	.01	[–.01,.02]
Percentage telework⪢work pressure⪢employability	.01	.01	[–.00,.03]
Percentage telework⪢social support⪢employability	.01	.01	[–.00,.03]
Percentage telework⪢communication⪢employability	.02	.01	[–.00,.03]
Percentage telework⪢role clarity⪢employability	.05	.02	[.01,.08]^ *^

## Appendix 2

**Table A2:** Modification indices for the initial model of the relationship between telework, job characteristics, and sustainable employability outcomes

Suggested path	Modification index	Expected parameter change
Employability⪢percentage telework	18,94	–7,02
Employability⪢working hours	18,55	0,26
Duration of sick leave⪢percentage telework	16,09	5,56
Duration of sick leave⪢work pressure	16,01	0,49
Duration of sick leave⪢working hours	15,68	–0,20
Duration of sick leave⪢role clarity	12,01	–2,63
Working hours⪢duration of sick leave	11,19	–0,15
Working hours⪢employability	9,30	0,11
Vitality⪢working hours	7,63	0,15
Vitality⪢percentage telework	6,36	–3,42
Employability⪢role clarity	6,03	1,01
Employability⪢work pressure	5,22	–0,18
Duration of sick leave⪢social support	4,26	2,93
Duration of sick leave⪢communication	3,88	2,85
Vitality⪢work pressure	3,82	–0,19
Vitality⪢role clarity	2,45	0,48
Employability⪢social support	1,14	–0,42
Employability⪢communication	1,04	–0,40
Frequency of sick leave⪢work pressure	0,93	0,12
Working hours⪢frequency of sick leave	0,59	0,04
Vitality⪢social support	0,57	–0,21
Frequency of sick leave⪢percentage telework	0,54	1,07
Vitality⪢communication	0,52	–0,20
Frequency of sick leave⪢working hours	0,47	–0,04
Working hours⪢vitality	0,43	0,03
Work pressure⪢role clarity	0,42	0,03
Frequency of sick leave⪢role clarity	0,42	–0,53
Social support⪢work pressure	0,36	–0,03
Communication⪢work pressure	0,33	–0,03
Frequency of sick leave⪢social support	0,33	1,30
Frequency of sick leave⪢communication	0,32	1,36
Work pressure⪢social support	0,20	–0,02
Work pressure⪢communication	0,19	–0,02
Role clarity⪢work pressure	0,11	0,02
Work pressure⪢percentage telework	0,00	0,00
Social support⪢percentage telework	0,00	0,00
Role clarity⪢percentage telework	0,00	0,00
Working hours⪢percentage telework	0,00	0,00
Work pressure⪢working hours	0,00	0,00
Social support⪢working hours	0,00	0,00
Role clarity⪢working hours	0,00	0,00
Percentage telework⪢working hours	0,00	0,00

In selecting which additional paths to include in our improved model, we first examined the suggested paths with a modification index of at least 5. We then assessed whether these suggested paths could be deemed reasonable according to the literature referenced by our study. In selecting additional paths, we also attempted to preserve the hypothesised order of our variables: that work pressure is linked to job demands and job resources, which in turn is linked to sustainable employability outcomes. Based on these considerations, and after deliberation between authors, working hours was included as a predictor of both duration of sick leave and employability in the improved model. As the inclusion of these two additional paths yielded an adequate fit in the improved model, no further modifications were made.

## References

[bibr1-WOR-240033] Kingma S . New ways of working (NWW): Work space and cultural change in virtualizing organizations. Cult Organ. (2019;25:383–406. 10.1080/14759551.2018.1427747

[bibr2-WOR-240033] Sullivan C . What’s in a name? Definitions and conceptualisations of teleworking and homeworking. New Technol Work Employ. 2003;18:158–65. 10.1111/1468-005X.00118

[bibr3-WOR-240033] Raišienėq AG Rapuano V Dőry T Varkulevičiūtėq K . Does telework work? Gauging challenges of telecommuting to adapt to a “new normal.”. Hum Technol. 2021;17:126–44. 10.14254/1795-6889.2021.17-2.3

[bibr4-WOR-240033] Barrero JM Bloom N Davis S . Why working from home will stick. Cambridge, MA: National Bureau of Economic Research; 2021. 10.3386/w28731

[bibr5-WOR-240033] Bollestad V Amland J-S Olsen E . The pros and cons of remote work in relation to bullying, loneliness and work engagement: A representative study among Norwegian workers during COVID-19. Front Psychol. 2022;13:1016368. 10.3389/fpsyg.2022.1016368 36389502 PMC9641189

[bibr6-WOR-240033] Bamieh O Ziegler L . Are remote work options the new standard? Evidence from vacancy postings during the COVID-19 crisis. Labour Econ. 2022;76:102179. 10.1016/j.labeco.2022.102179 35578707 PMC9093064

[bibr7-WOR-240033] Valdez ID Ramirez CE Khansari A Momin I Sitzmann C Dehghanpour M , et al. Leading past COVID- An analysis of remote work now and beyond. Med Dosim. 2023;48:161–4. 10.1016/j.meddos.2023.03.003 37062599 PMC10033252

[bibr8-WOR-240033] Rijksoverheid. AOW leeftijd stijgt minder snel. Rijksoverheid. 2021. https://www.rijksoverheid.nl/onderwerpen/pensioen/toekomst-pensioenstelsel/aow-leeftijd-stijgt-minder-snel (accessed May 16, 2021).

[bibr9-WOR-240033] Van Der Heijden B . No one has ever promised you a rose garden, 2006.

[bibr10-WOR-240033] De Lange A Van Der Heijden B . Een leven lang inzetbaar? Duurzame inzetbaarheid op het werk: Interventies, best practices en integrale benaderingen. 2nd ed. Vakmedianet; 2016.

[bibr11-WOR-240033] Van Der Klink JJ Bültmann U Burdorf A Schaufeli WB Zijlstra FR Abma FI , et al. Sustainable employability – definition, conceptualization, and implications: A perspective based on the capability approach. Scand J Work Environ Health. 2016;42:71–9. 10.5271/sjweh.3531 26595874

[bibr12-WOR-240033] Collins AM Hislop D Cartwright S . Social support in the workplace between teleworkers, office-based colleagues and supervisors. New Technol Work Employ. 2016;31:161–75. 10.1111/ntwe.12065

[bibr13-WOR-240033] Wöhrmann AM Ebner C . Understanding the bright side and the dark side of telework: An empirical analysis of working conditions and psychosomatic health complaints. New Technol Work Employ. 2021;36:348–70. 10.1111/ntwe.12208

[bibr14-WOR-240033] Demerouti E Bakker AB Nachreiner F Schaufeli WB . The job demands-resources model of burnout. J Appl Psychol. 2001;86:499–512. 10.1037/0021-9010.86.3.499 11419809

[bibr15-WOR-240033] Bakker AB Demerouti E Sanz-Vergel A . Job demands–resources theory: Ten years later. Annu Rev Organ Psychol Organ Behav. 2023;10:25–53. 10.1146/annurev-orgpsych-120920-053933

[bibr16-WOR-240033] Boyd CM Bakker AB Pignata S Winefield AH Gillespie N Stough C . A Longitudinal Test of the Job Demands-Resources Model among Australian University Academics: A LONGITUDINAL TEST OF THE JD-R MODEL. Appl Psychol. 2011;60:112–40. 10.1111/j.1464-0597.2010.00429.x

[bibr17-WOR-240033] Schaufeli WB Bakker AB . Job demands, job resources, and their relationship with burnout and engagement: A multi-sample study. J Organ Behav. 2004;25:293–315. 10.1002/job.248

[bibr18-WOR-240033] Schaufeli W Taris T . Het Job Demands-Resources model: Overzicht en kritische beschouwing. Gedrag Organ. 2013;26. 10.5117/2013.026.002.182

[bibr19-WOR-240033] Sardeshmukh SR Sharma D Golden TD . Impact of telework on exhaustion and job engagement: A job demands and job resources model. New Technol Work Employ. 2012;27:193–207. 10.1111/j.1468-005X.2012.00284.x

[bibr20-WOR-240033] Deci EL Ryan RM . The general causality orientations scale: Self-determination in personality. J Res Personal. 1985;19:109–34. 10.1016/0092-6566(85)90023-6

[bibr21-WOR-240033] Barello S Caruso R Palamenghi L Nania T Dellafiore F Bonetti L , et al. Factors associated with emotional exhaustion in healthcare professionals involved in the COVID-19 pandemic: An application of the job demands-resources model. Int Arch Occup Environ Health. 2021;94:1751–61. 10.1007/s00420-021-01669-z 33660030 PMC7928172

[bibr22-WOR-240033] Bilotta I Cheng S Davenport MK King E . Using the job demands-resources model to understand and address employee well-being during the COVID-19 pandemic. Ind Organ Psychol. 2021;14:267–73. 10.1017/io2021.43

[bibr23-WOR-240033] Zhou T Xu C Wang C Sha S Wang Z Zhou Y , et al. Burnout and well-being of healthcare workers in the post-pandemic period of COVID- A perspective from the job demands-resources model. BMC Health Serv Res. 2022;22:284. 10.1186/s12913-022-07608-z 35236354 PMC8888816

[bibr24-WOR-240033] Hamouche S . Human resource management and the COVID-19 crisis: Implications, challenges, opportunities, and future organizational directions. J Manag Organ. 2023;29:799–814. 10.1017/jmo.2021.15

[bibr25-WOR-240033] Bailey DE Kurland NB . A review of telework research: Findings, new directions, and lessons for the study of modern work. J Organ Behav. 2002;23:383–400. 10.1002/job.144

[bibr26-WOR-240033] Harker Martin B MacDonnell R . Is telework effective for organizations?: A meta-analysis of empirical research on perceptions of telework and organizational outcomes. Manag Res Rev. 2012;35:602–16. 10.1108/01409171211238820

[bibr27-WOR-240033] Fleuren BB De Grip A Jansen NW Kant I Zijlstra FR . Critical reflections on the currently leading definition of sustainable employability. Scand J Work Environ Health. 2016;42:557–60. 10.5271/sjweh.3585 27548816

[bibr28-WOR-240033] Jabeen Q Nadeem MS Raziq MM Sajjad A . Linking individuals’ resources with (perceived) sustainable employability: Perspectives from conservation of resources and social information processing theory. Int J Manag Rev. 2022;24:233–54. 10.1111/ijmr.12276

[bibr29-WOR-240033] Magnano P Santisi G Zammitti A Zarbo R Di Nuovo S . Self-perceived employability and meaningful work: The mediating role of courage on quality of life. Sustainability. 2019;11:764. 10.3390/su11030764

[bibr30-WOR-240033] Semeijn JH Caniëls MCJ Kooistra D . Cross-lagged effects of resilience and indicators of sustainable employability; a study among Dutch police officers. Polic Int J. 2019;42:961–75. 10.1108/PIJPSM-01-2019-0003

[bibr31-WOR-240033] Schaufeli W Bakker A , editors. De psychologie van arbeid en gezondheid. Houten: Bohn Stafleu van Loghum; 2020. 10.1007/978-90-368-2495-8

[bibr32-WOR-240033] Ilmarinen J Tuomi K Seitsamo J . New dimensions of work ability. Int Congr Ser. 2005;1280:3–7. 10.1016/j.ics.2005.02.060

[bibr33-WOR-240033] Van Der Heijde C Van Der Heijden BIJM . A competence-based and multidimensional operationalization and measurement of employability. Hum Resour Manage. 2006;45:449–76. 10.1007/978-90-368-2495-8

[bibr34-WOR-240033] Stoffers JM Van Der Heijden BIJM Notelaers GLA . Towards a moderated mediation model of innovative work behaviour enhancement. J Organ Change Manag. 2014;27:642–59. 10.1108/JOCM-05-2014-0105

[bibr35-WOR-240033] Irfan SM Qadeer F Abdullah MI Sarfraz M . Employer’s investments in job crafting to promote knowledge worker’s sustainable employability: A moderated mediation model. Pers Rev. 2023;52:2025–47. 10.1108/PR-10-2021-0704

[bibr36-WOR-240033] Ybema JF Van Vuuren T Van Dam K . HR practices for enhancing sustainable employability: Implementation, use, and outcomes. Int J Hum Resour Manag. 2020;31:886–907. 10.1080/09585192.2017.1387865

[bibr37-WOR-240033] Meerman J Van Casteren PAJ Brouwers EPM Van Dam A Van Der Klink JJL . A capability perspective on sustainable employability: A Dutch focus group study on organizational, work and personal conversion factors. PLOS ONE. 2022;17:e0274000. 10.1371/journal.pone.0274000 PMC9586339 36269699

[bibr38-WOR-240033] Roe RA Zijlstra FRH . Work pressure. Results of a conceptual and empirical analysis 2000.

[bibr39-WOR-240033] Jamal MT Anwar I Khan NA Saleem I . Work during COVID- Assessing the influence of job demands and resources on practical and psychological outcomes for employees. Asia-Pac J Bus Adm. 2021;13:293–319.

[bibr40-WOR-240033] Bekkers H . Stressen in coronatijd? Stop op tijd. Binnelands Best 2021. 10.1108/APJBA-05-2020-0149

[bibr41-WOR-240033] Zhang S Moeckel R Moreno AT Shuai B Gao J . A work-life conflict perspective on telework. Transp Res Part Policy Pract. 2020;141:51–68. 10.1016/j.tra.2020.09.007 PMC7509537 32982087

[bibr42-WOR-240033] Pearson JE . The definition and measurement of social support. J Couns Dev. 1986;64:390–5. 10.1002/j.1556-6676.1986.tb01144.x

[bibr43-WOR-240033] Ye Z Yang X Zeng C Wang Y Shen Z Li X , et al. Resilience, social support, and coping as mediators between COVID-19-related stressful experiences and acute stress disorder among college students in China. Appl Psychol Health Well-Being. 2020;12:1074–94. 10.1111/aphw.12211 32666713 PMC7405224

[bibr44-WOR-240033] Brouwers A Evers WJG Tomic W . Self-efficacy in eliciting social support and burnout among secondary-school teachers. J Appl Soc Psychol. 2001;31:1474–91. 10.1111/j.1559-1816.2001.tb02683.x

[bibr45-WOR-240033] Vanheule S Declercq F Meganck R Desmet M . Burnout, critical incidents and social support in security guards. Stress Health. 2008;24:137–41. 10.1002/smi.1177

[bibr46-WOR-240033] Schaufeli W Le Blanc P . Theoretische modellen over werkstress. In: Schaufeli W Bakker A , editors. Psychol. Van Arb. En Gezondh., Houten: Bohn Stafleu van Loghum; 2020, p. 25–47. 10.1007/978-90-368-2495-8_2

[bibr47-WOR-240033] Jolly PM Kong DT Kim KY . Social support at work: An integrative review. J Organ Behav. 2021;42:229–51. 10.1002/job.2485.

[bibr48-WOR-240033] Xia T Gu H Huang Y Zhu Q Cheng Y . The relationship between career social support and employability of college students: A moderated mediation model. Front Psychol. 2020;11:28. 10.3389/fpsyg.2020.00028 32047461 PMC6997469

[bibr49-WOR-240033] Jiang Z . Social support and career psychological states: An integrative model of person– environment fit. J Career Assess. 2017;25:219–37. 10.1177/1069072715621019

[bibr50-WOR-240033] Bekkers H . Plus- en minpunten van thuiswerken. Binnelands Best 2020.

[bibr51-WOR-240033] Schneider FM Maier M Lovrekovic S Retzbach A . The Perceived Leadership Communication Questionnaire (PLCQ): Development and validation. J Psychol. 2015;149:175–92. 10.1080/00223980.2013.864251 25511204

[bibr52-WOR-240033] Motschnig R Ryback D . Transforming Communication in Leadership and Teamwork. Cham: Springer International Publishing; 2016. 10.1007/978-3-319-45486-3

[bibr53-WOR-240033] Arnold JA Arad S Rhoades JA Drasgow F . The empowering leadership questionnaire: The construction and validation of a new scale for measuring leader behaviors. J Organ Behav. 2000;21:249–69.

[bibr54-WOR-240033] Watson-Manheim MB Piramuthu S Narasimhan S . Exploratory analysis of factors influencing performance dynamics of telecommuters and traditional office workers. IEEE Trans Syst Man Cybern Part C Appl Rev. 2000;30:239–51. 10.1109/5326.868445

[bibr55-WOR-240033] Lynn G Kalay F . The effect of vision and role clarity on team performance. J Bus Econ Finance. 2015;4:473–473. 10.17261/Pressacademia.2015313067

[bibr56-WOR-240033] Bakker AB Petrou P Op Den Kamp EM Tims M . Proactive Vitality Management, Work Engagement, and Creativity: The Role of Goal Orientation. Appl Psychol. 2020;69:351–78. 10.1111/apps.12173

[bibr57-WOR-240033] Bakker AB Schaufeli WB . Positive organizational behavior: Engaged employees in flourishing organizations. J Organ Behav. 2008;29:147–54. 10.1002/job.515

[bibr58-WOR-240033] Puyod JV Charoensukmongkol P . Effects of workplace rumors and organizational formalization during the COVID-19 pandemic: A case study of universities in the Philippines. Corp Commun Int J. 2021;26:793–812. 10.1108/CCIJ-09-2020-0127

[bibr59-WOR-240033] Mühlenbrock I Richter G Ellerkamp A Wöhrmann AM . How does telework modify informal workplace learning and how can supervisors provide support? Gr Interakt Organ Z Für Angew Organ GIO. 2023;54:311–21. 10.1007/s11612-023-00692-7

[bibr60-WOR-240033] TNO. Nationale Enquête Arbeidsomstandigheden voor Werknemers 2019.

[bibr61-WOR-240033] Rizzo JR House RJ Lirtzman SI . Role conflict and ambiguity in complex organizations. Adm Sci Q. 1970;15:150. 10.2307/2391486

[bibr62-WOR-240033] Schaufeli WB Bakker AB . Bevlogenheid: Een begrip gemeten. Gedrag Organ. 2004;17. 10.5117/2004.017.002.002

[bibr63-WOR-240033] Van Vuuren T Stoffers J Lancée V . Het effect van opleiding en training op de duurzame inzetbaarheid van medewerkers: Een longitudinale studie op grond van objectieve data. Tijdschr Voor HRM. 2018;21:18–35. 10.5117/THRM2018.1.VUUR

[bibr64-WOR-240033] Van Der Heijden BIJM Notelaers G Peters P Stoffers JMM De Lange AH Froehlich DE , et al. Development and validation of the short-form employability five-factor instrument. J Vocat Behav. 2018;106:236–48. 10.1016/j.jvb.2018.02.003

[bibr65-WOR-240033] Crawford J . Working from home, telework, and psychological wellbeing? A systematic review. Sustainability. 2022;14:11874. 10.3390/su141911874

[bibr66-WOR-240033] Lopes S Dias PC Sabino A Cesário F Peixoto R . Employees’ fit to telework and work well-being: (in)voluntariness in telework as a mediating variable?. Empl Relat Int J. 2023;45:257–74. 10.1108/ER-10-2021-0441

[bibr67-WOR-240033] Iacobucci D . Structural equations modeling: Fit Indices, sample size, and advanced topics. J Consum Psychol. 2010;20:90–8. 10.1016/j.jcps.2009.09.003

[bibr68-WOR-240033] Van Hooff MLM Van Hooft EAJ . Boredom at work: Towards a dynamic spillover model of need satisfaction, work motivation, and work-related boredom. Eur J Work Organ Psychol. 2017;26:133–48. 10.1080/1359432X.2016.1241769

[bibr69-WOR-240033] Van Stralen M Noordik E Roelen C . Persoons-en ziektegebonden factoren geassocieerd met de verzuimduur na COVID-19. TBV – Tijdschr Voor Bedr- En Verzek. 2021;29:18–23. 10.1007/s12498-021-1406-2 PMC8190747 34127885

[bibr70-WOR-240033] Ruhle SA Schmoll R . COVID-19, telecommuting, and (virtual) sickness presenteeism: Working from home while ill during a pandemic. Front Psychol. 2021;12:734106. 10.3389/fpsyg.2021.734106 34721202 PMC8554096

[bibr71-WOR-240033] Bharadwaj P Pai M Suziedelyte A . Mental health stigma. Cambridge, MA: National Bureau of Economic Research; 2015. 10.3386/w21240

[bibr72-WOR-240033] Taris TW Kessler SR Kelloway EK . Strategies addressing the limitations of cross-sectional designs in occupational health psychology: What they are good for (and what not). Work Stress. 2021;35:1–5. 10.1080/02678373.2021.1888561

[bibr73-WOR-240033] Fiedler K Harris C Schott M . Unwarranted inferences from statistical mediation tests – An analysis of articles published in 2015. J Exp Soc Psychol. 2018;75:95–102. 10.1016/j.jes2017.11.008

[bibr74-WOR-240033] Dormann C Griffin MA . Optimal time lags in panel studies. Psychol Methods. 2015;20:489–505. 10.1037/met0000041 26322999

[bibr75-WOR-240033] Lesener T Gusy B Wolter C . The job demands-resources model: A meta-analytic review of longitudinal studies. Work Stress. 2019;33:76–103. 10.1080/02678373.2018.1529065

[bibr76-WOR-240033] LeBlanc VR . The effects of acute stress on performance: Implications for health professions education. Acad Med. 2009;84:S25–33. 10.1097/ACM.0b013e3181b37b8f 19907380

[bibr77-WOR-240033] Massad CM Hubbard M Newtson D . Selective perception of events. J Exp Soc Psychol. 1979;15:513–32. 10.1016/0022-1031(79)90049-0

[bibr78-WOR-240033] Rampersad H Hussain S . Personal Balanced Scorecard. Authentic Gov., Cham: Springer International Publishing; 2014, p. 29–38. 10.1007/978-3-319-03113-2_4

[bibr79-WOR-240033] Bauer TN Erdogan B . Organizational socialization: The effective onboarding of new employees. In: Zedeck S , editor. APA Handb. Ind. Organ. Psychol. Vol 3 Maint. Expand. Contract. Organ., Washington: American Psychological Association; 2011, p. 51–64. 10.1037/12171-002

[bibr80-WOR-240033] Dekker F Koster F . Thuiswerken en innovatie: Het gaat er niet om waar je werkt. Mens Maatsch. 2020;95:321–37. 10.5117/MEM2020.4.002.DEKK

